# Assessment of the fetal thymus gland: Comparing MRI-acquired thymus volumes with 2D ultrasound measurements

**DOI:** 10.1016/j.ejogrb.2021.06.026

**Published:** 2021-06-30

**Authors:** Rebecca Myers, Jana Hutter, Jacqueline Matthew, Tong Zhang, Alena Uus, David Lloyd, Alexia Egloff, Maria Deprez, Surabhi Nanda, Mary Rutherford, Lisa Story

**Affiliations:** a King’s College London School of Bioscience, St George’s, University of London, UK; b Department of Perinatal Imaging, School of Biomedical Engineering, King’s College London, UK; c Artificial Intelligence Research Center, Peng Cheng Laboratory, Shenzhen, China; d Department of Fetal Medicine, St Thomas’ Hospital London, UK; e Department of Women and Children’s Health King’s College London, UK

**Keywords:** Fetal thymus, Magnetic resonance imaging, Obstetric ultrasound thymus, Volume

## Abstract

**Objectives:**

The fetal thymus gland has been shown to involute in response to intrauterine infection, and therefore could be used as a non-invasive marker of fetal compartment infection. The objective of this study was to evaluate how accurately 2D ultrasound-derived measurements of the fetal thymus reflect the 3D volume of the gland derived from motion corrected MRI images.

**Study design:**

A retrospective study was performed using paired ultrasound and MRI datasets from the iFIND project (http://www.ifindproject.com). To obtain 3D volumetry of the thymus gland, T2-weighted single shot turbo spin echo (ssTSE) sequences of the fetal thorax were acquired. Thymus volumes were manually segmented from deformable slice-to-volume reconstructed images. To obtain 2D ultrasound measurements, previously stored fetal cine loops were used and measurements obtained at the 3-vessel-view (3VV) and 3-vessel-trachea view (3VT): anterior-posterior diameter (APD), intrathoracic diameter (ITD), transverse diameter (TD), perimeter and 3-vessel-edge (3VE). Inter-observer and intra-observer reliability (ICC) was calculated for both MRI and ultrasound measurements. Pearson correlation coefficients (PCC) were used to compare 2D-parameters with acceptable ICC to TV.

**Results:**

38 participants were identified. Adequate visualisation was possible on 37 MRI scans and 31 ultrasound scans. Of the 30 datasets where both MRI and ultrasound data were available, MRI had good interobserver reliability (ICC 0.964) and all ultrasound 3VV 2D-parameters and 3VT 3VE had acceptable ICC (>0.75). Four 2D parameters were reflective of the 3D thymus volume: 3VV TD *r* = 0.540 (P = 0.002); 3VV perimeter *r* = 0.446 (P = 0.013); 3VV APD *r* = 0.435 (P = 0.110) and 3VT TD *r* = 0.544 (P = 0.002).

**Conclusions:**

MRI appeared superior to ultrasound for visualization of the thymus gland and reproducibility of measurements. Three 2D US parameters, 3VV TD, perimeter and 3VT APD, correlated well with TV. Therefore, these represent a more accurate reflection of the true size of the gland than other 2D measurements, where MRI is not available.

## Introduction

The fetal thymus is a primary lymphoid organ involved in the development and differentiation of T-cells as part of the fetal immune system [1]. The fetal thymus has also been demonstrated to involute in response to intrauterine infection in pregnancies affected by preterm premature rupture of membranes (PPROM) [2,3]. These findings suggest that the fetal thymus gland may be an early, non-invasive indicator of fetal compartment infection in high-risk pregnancies.

Currently, there is no direct, non-invasive method to identify fetal compartment infection and clinical markers, including elevated maternal temperature, maternal and fetal tachycardia and uterine tenderness, are used in conjunction with raised maternal inflammatory markers to make this diagnosis [2,4]. However, cases of fetal compartment infection, such as those associated with PPROM, have been shown to present without overt clinical signs [5,6], suggesting that by the time of presentation, a fetal infection may already be established. In addition, adverse infant outcomes including bronchopulmonary dysplasia [2,7,8], cerebral palsy [9], intraventricular haemorrhage [10] and neonatal sepsis and pneumonia [2] have been associated with the presence of intrauterine infection. The most significant adverse neonatal outcomes occur in fetuses that deliver very prematurely (before 32 weeks gestation) [8,10].

Although antenatal ultrasound (US) has been successfully used to identify the fetal thymus gland [11–13], US data is often limited by adverse fetal positioning [12,13], increased maternal body habitus [14] and oligohydramnios [15]. Furthermore, the two-dimensional (2D) measurements used in these studies may not account for the inherent variability of the three-dimensional (3D) thymus gland.

3D imaging of the fetal thymus on antenatal US has also been attempted and has been shown to provide improved visualisation of its borders [16]. However, the technique is time-consuming [17], limited by both acoustic shadowing and movement artefact [18] and has poor inter-observer reliability [19]. MRI is less sensitive to fetal lie and increased maternal habitus, and slice-to-volume reconstruction methods can account for unpredictable fetal motion [20] providing a more accurate representation of true fetal thymic size [21]. Fetal thymus volume (TV) has been successfully measured on fetal magnetic resonance imaging (MRI) in both normal and growth-restricted fetuses [22] and those at high risk of preterm birth [21]. However, the cost of MRI in comparison to conventional ultrasound may preclude its use in routine clinical practice.

This study therefore aims to assess the suitability of two-dimensional US-derived measures of thymus size as a proxy marker for overall size of the gland, measured from motion corrected MRI datasets, in fetuses between 20^+0^ and 32^+0^ weeks gestation by:

1. Assessing the reproducibility of 2D US and 3D MRI measurements of the fetal thymus
2. Comparing 2D US measurements with 3D MRI-derived TV

## Materials and methods

### Participants

Datasets had previously been acquired as part of the intelligent fetal imaging and diagnosis (iFIND) project (http://www.ifindproject.com). Cases were selected in singleton pregnancies when: both US and MRI scanning had been undertaken between 20^+0^ and 32^+0^ weeks gestation; MRI scanning had occurred on a 1.5 T MRI system; no antenatal complications had occurred; and pregnancies were delivered after 37^+0^ weeks gestation. All women had undergone US and MRI scanning within a 3-day period. Maternal demographics were recorded.

### MRI scans

All women had given informed, written consent (Ethics reference 14/LO/1806). All fetal MRI were performed on a 1.5 T Philips Ingenia MRI system (Philips Medical systems, Best, the Netherlands) with a 28-channel Torso-coil placed on the mother’s abdomen. The mother was scanned in the “left lateral tilt” position. Imaging of the fetus was performed using T2-weighted single shot turbo spin echo (ssTSE), obtained in three orthogonal planes. The following scanning parameters were followed: TR = 25,991 ms, TE = 80 ms, slice thickness = 2.5 mm, slice overlap = 1.25 mm and flip angle 90°. Medical cover, by either an obstetrician or midwife, frequent verbal interaction with mother and continuous monitoring of oxygen saturation and heart rate was provided throughout the scan. Scanning time was limited to one hour.

The fetal thorax was reconstructed in order to correct motion-artefacts. Slice-to-volume reconstruction tool (SVR) was performed, using 6–8 MRI stacks acquired in different directions through the fetal thorax [23,24]. Using the software ITK-SNAP version 3.6.0 [25], the fetal thymus was manually segmented from the SVR volumes, enabling the acquisition of thymus volume from the fetal thorax. All segmentations were undertaken by one operator (LS). Good inter observer variability had previously been confirmed with a further operator (AE) [21].

### Ultrasound scans

All fetal ultrasounds were performed on a Philips EPiQ ultrasound system (Best, Netherlands) with a high frequency (5–9 mHz) curvilinear probe. A dedicated fetal cardiac preset was used. The acquisition plane was achieved by an experienced fetal cardiologist or senior obstetric sonographer [26]. Five second cine loops were stored of all cardiac imaging when the plane of interest was achieved.

**Fig. 1:**
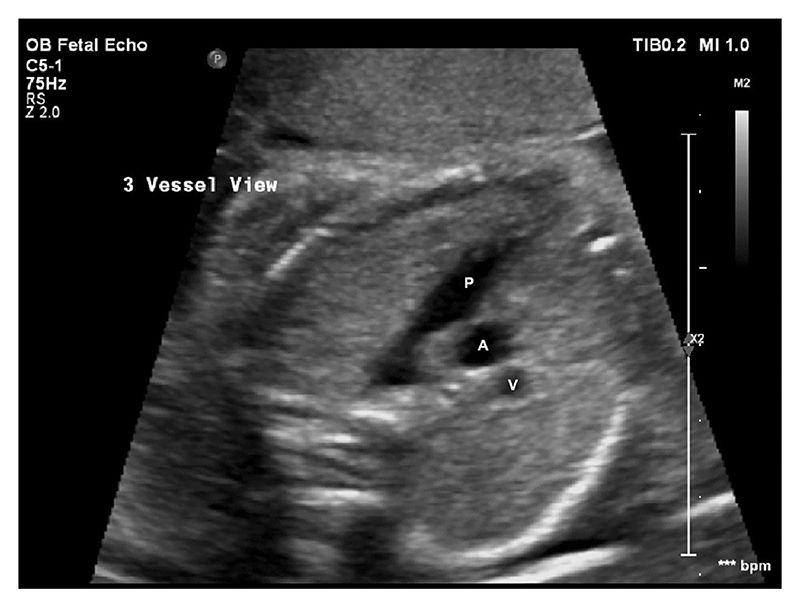
Obstetric ultrasound of fetal thorax showing the 3-vessel-view (3VV). Pulmonary Artery (P), ascending aorta (A) and superior vena cava (V).

**Fig. 2:**
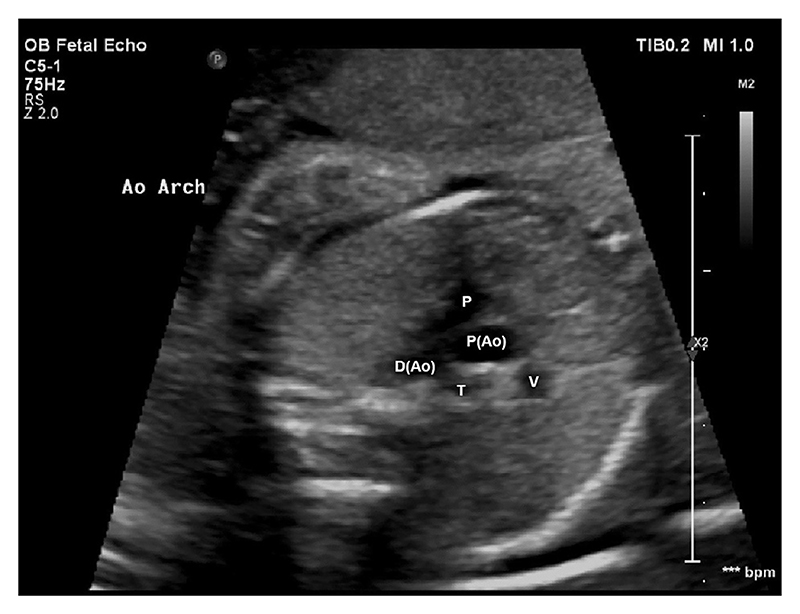
Obstetric ultrasound of fetal thorax in the 3-vessel-trachea view (3VT). Pulmonary artery (P), proximal aorta (P(Ao)), distal aorta (D(Ao)), superior vena cava (V), trachea (T).

Previously cited 2D measurements, were obtained from the recorded cine loops using Medical Imaging Interaction Toolkit (MITK) Workbench software (version 2018.04.2) [27] at two anatomical levels: the 3-vessel-view (3VV) shown in Fig. 1, and the 3-vessel-trachea view (3VT) shown in Fig. 2 [26].

**Fig. 3:**
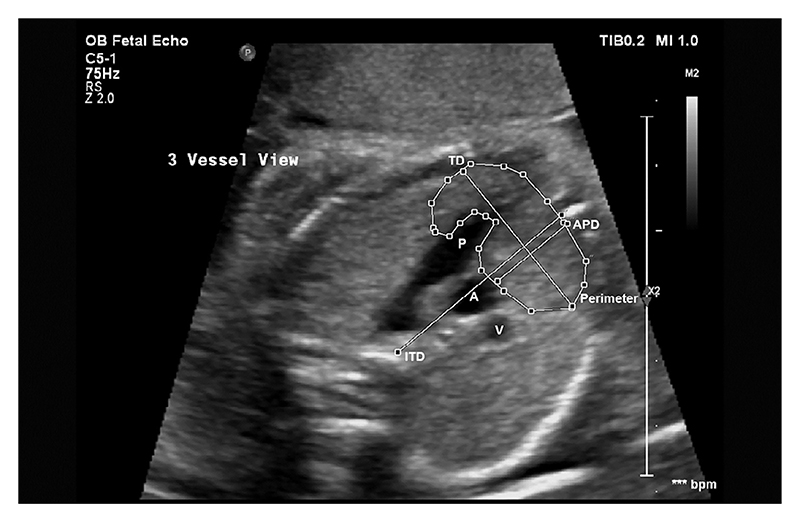
Obstetric ultrasound of fetal thorax showing the 3-vessel-view (3VV). Pulmonary Artery (P), ascending aorta (A) and superior vena cava (V), Anterior-Posterior Diameter (APD), Intrathoracic Diameter (ITD), Transverse Diameter (TD) and Perimeter.

**Fig. 4:**
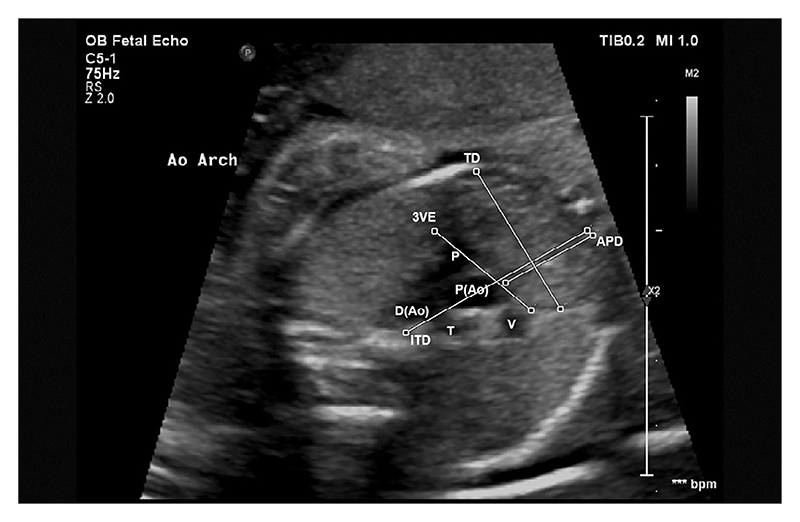
Obstetric ultrasound of fetal thorax showing the 3-vessel-trachea view (3VT). Pulmonary artery (P), proximal aorta (P(Ao)), distal aorta (D(Ao)), superior vena cava (V), trachea (T), Anterior-Posterior Diameter (APD), Intrathoracic Diameter (ITD), Transverse Diameter (TD) and 3-vessel-edge (3VE).

At the 3VV, as in Chaoui et al [28], the thymic-thoracic ratio (TTR) was calculated by dividing the anterior-posterior diameter (APD) of the thymus by the intrathoracic diameter (ITD). The maximum transverse diameter (TD) was then measured perpendicularly to the ITD at the widest point of the thymus gland, as in Cho et al [13]. Perimeter was measured through manual tracing of the thymic borders, as described in Zalel et al [12]. This is shown in Fig. 3. At the 3VT, APD, ITD and TD were measured, as well as 3-vessel-edge (3VE), which was measured by drawing a straight line through the anterior borders of the superior vena cava (SVC), proximal aortic arch and pulmonary artery (PA), as shown by Diemert et al [17]. This is shown in Fig. 4.

For all 2D measurements, inter observer reliability was determined between four operators, including a fetal medicine consultant, senior radiographer and fetal cardiologist (SN, JM, DL and RM) across 5 control scans. Intra-observer reliability was also confirmed (RM). Maternal demographics and neonatal parameters were recorded. All datasets were then analysed by one operator (RM).

### Statistical analysis

Intraclass correlation coefficients (ICC) were calculated to obtain the intra- and inter-observer reliability for 2D US-derived measurements of the thymus gland in each of the above views. Intra- and inter-observer variability of MRI-derived TV had previously been ascertained [21]. Pearson correlation coefficients (PCC) (*r*) were then used to compare US-derived measurements with the 3D MRI-derived TV. Analyses were conducted using IBM SPSS Statistics Version 26.0.

## Results

### Participants

During the period of the study, 38 cases were selected from existing datasets (iFIND project). On fetal MRI, 3D reconstruction of the fetal thorax was completed in all women. The fetal thymus could not be visualised in one case. In US imaging, visualisation of the thymus gland was not possible in seven cases (18%) due to acoustic shadowing impeding visualisation of the thymus borders. This left 30 cases with both US and MRI images suitable for comparison. All fetuses had normal liquor volume on scan.

Maternal and neonatal parameters were recorded. Mean maternal age was 32.83 years (range (R) = 20–39 years) and mean maternal BMI was 22.9 kg/m^2^ (R = 19–33 kg/m^2^).

### Thymic measurements

Intra and inter-observer reliability was calculated using ICC are presented in Table 1. Coefficients > 0.75 were accepted as an indicator of good reliability [29]. Intra-observer reliability for MRI-derived TV was confirmed previously between two expert operators (AE and LS), ICC = 0.964, P<=0.0001) [21].

Pearson correlation coefficients (PCC) (*r* and *r*^2^) were used to compare 2D US measurements with TV and are presented in Table 2. 3VV APD, TD, and perimeter, as well as 3VT TD correlated with TV and were statistically significant (P<=0.05), whereas 3VV ITD, TTR and 3VT ITD, TTR and 3VE were neither well correlated nor statistically significant [30].

## Discussion

### Reproducibility of thymic measurements

MRI proved superior for visualisation of the thymus gland, with adequate visualisation occurring in 97% of cases, compared to 80% (30/38) on ultrasound. MRI-derived TV also provided the most reproducible method of assessment of thymus size (0.964, P < 0.01) [21].

Few studies have assessed the fetal thymus using MRI, however a previous study from our research group evaluated the gland in 39 fetuses with the gland visualised in all cases [22]. Although some studies have reported poor visualisation of the fetal thymus on ultrasound [11,18], our finding that adequate visualisation occurred in only 80% of cases on ultrasound images is lower than previously reported studies where rates of up to 100% have been described [12,19]. All previous studies were conducted by specialists specifically assessing the thymus gland. However, our datasets were evaluated retrospectively from stored cine loops, with measurements from two planes (3VV and 3VT-view), which have been previously described as necessary for obtaining optimal thymus images to ensure standardization of measurements [12,13,17,26,28]. Our results may therefore be more reflective of practitioners assessing the thymus in clinical practice.

In the present study, 2D measurements with acceptable inter-observer reliability (ICC > 0.75, P<=0.05) were 3VV APD (0.913), ITD (0.931), TD (0.872) and perimeter (0.788), as well as 3VT 3VE (0.913), with only 3VV APD and 3VT 3VE with excellent inter-observer reliability (>0.9) [29]. Multiple previous studies have reported good agreement for all 2D parameters [12,13,17,28], although Diemert et al found poor inter-observer reliability for thymic perimeter [17]. There are difficulties in obtaining standardised planar views on ultrasound imaging; 2D measurements are angle-dependent, meaning that only a few degrees of error can have significant effects on the overall measurement. Furthermore, ultrasound data was stored as cine loops and not observed in real time, meaning that investigators had a limited number of views to measure from. Therefore, these differences may be exacerbated in clinical practice without a preset number of frames to view. However, it is likely that US may perform better with prospective measurements as individual adjustments can be made to allow for better visualisation.

Furthermore, although acceptable ICC for MRI-derived TV has been identified in growth restricted fetuses previously [22], the high ICC obtained in the present study (0.964, P<=0.0001) is likely due to improvements in slice-to-volume reconstruction techniques [20].

### Comparison of 2D-ultrasound and 3D-MR thymus measurements

**Fig. 5:**
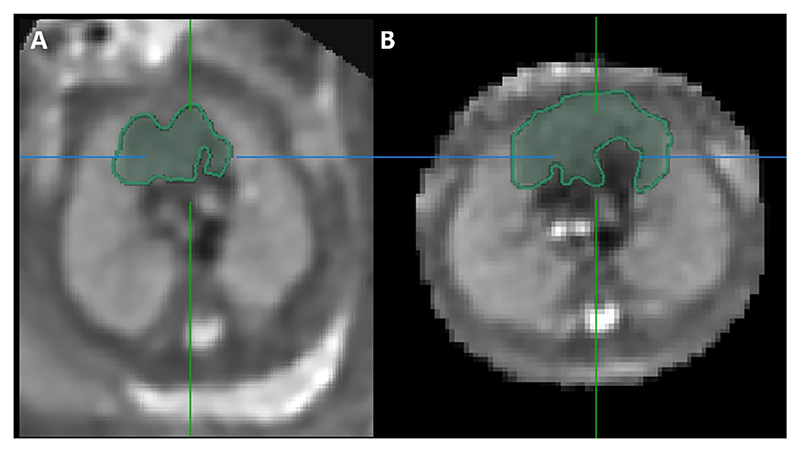
Slice-to-volume reconstructed MRI images of the fetal thorax from two fetuses showing the segmented fetal thymus gland in the axial plane. Scan A conducted at 24 + 6 weeks gestation, Scan B conducted at 24 + 2 weeks gestation.

**Fig. 6:**
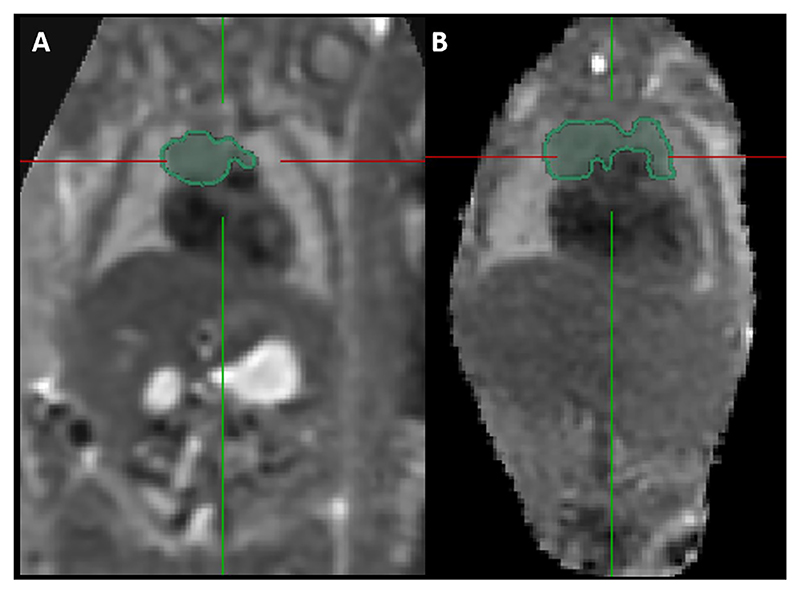
Slice-to-volume reconstructed MRI images of the fetal thorax from two fetuses showing the segmented fetal thymus gland in the coronal plane. Scan A conducted at 24 + 6 weeks gestation. Scan B conducted at 24 + 2 weeks gestation.

**Fig. 7:**
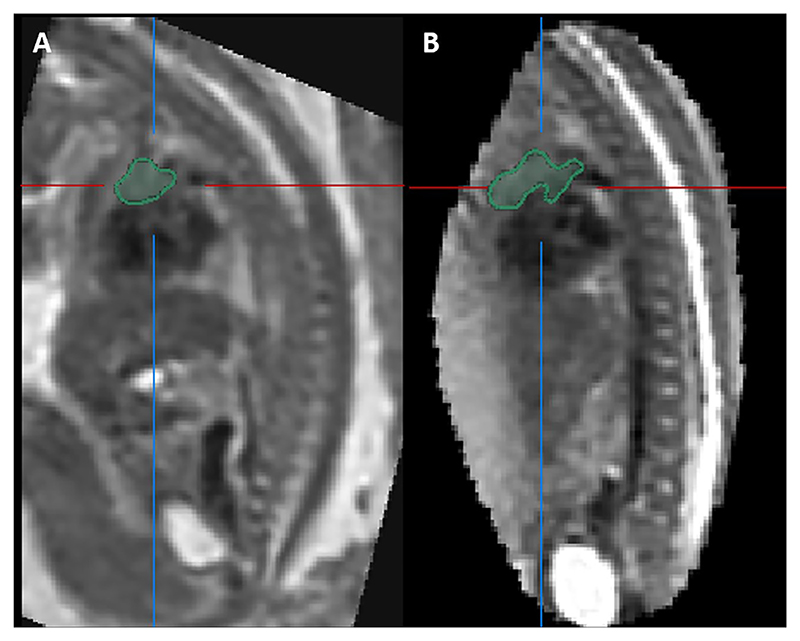
Slice-to-volume reconstructed MRI images of the fetal thorax from two fetuses showing the segmented fetal thymus gland in the sagittal plane. Scan A conducted at 24 + 6 weeks gestation. Scan B conducted at 24 + 2 weeks gestation.

Results from the study found only four 2D parameters correlated with MR-derived thymus volume [30]: 3VV TD, APD and perimeter, and 3VT TD. The variation in correlation between different 2D measurements and TV may be attributable to significant variability in thymus shape even within healthy fetuses [31], where some thymuses have a more globular appearance and others more diffuse. This is demonstrated in Figs. 5, 6 and 7. Furthermore, r^2^ values for the 2D-parameters that best correlate with MRI-derived TV remain low. This indicates that approximately only 20 to 30% of the variance in MRI TV can be explained, highlighting that the variable thymus shape is poorly captured by linear measurements.

These findings are supported by Li et al who previously compared the size and volume of the thymus by 2D (maximum transverse diameter (TD), maximum transverse area (A) and anterior-posterior diameter (APD) and superior-inferior diameter (SID)) and 3D ultrasound in 321 fetuses [16]. Results found that the correlation between thymus volume and gestation age was stronger than between each of the 2D measurements individually (r(TV) = 0.99 and r(TA) = 0.92, r(TD) = 0.88, r(APD) = 0.85 and r(SID) = 0.82)), suggesting that TV is more accurate than 2D measurements in determining thymic size.

Furthermore, Tonni et al compared ultrasound-derived transverse diameter and TV, identifying the thymus gland with the use of Doppler ultrasound to identify perithymic vessels in order to improve visualisation of the gland [19]. Across 100 women, a good correlation between transverse diameter and TV was reported (r = 0.58p < 0.001). However, the inter-observer reliability for TV was low (ICC = 0.57), suggesting some difficulty obtaining accurate US-derived 3D measurements.

With US being a quick, non-resource intensive imaging technique, questions of accessibility to fetal MRI and difficulties in post-imaging processing have been raised; however a recent study demonstrating the advances in MRI post-acquisition techniques ensures processing can be completed with relative ease in a less time-consuming manner [32].

### Limitations

Limitations of the present study include a small sample size (n = 30) which was retrospective in nature. Additionally, all patients had normal liquor volume and a normal maternal BMI (R = 19–33 kg/m^2^). As both oligohydramnios [15] and raised maternal BMI [14] reduce visualisation of fetal structures on ultrasound, quantification of the fetal thymus may be even more challenging in these scenarios. Finally, confidence intervals for intra and inter-reliability were wide, which may relate to the small sample number (n = 5), that these values were determined from.

In the future, further 2D US measurements of the fetal thymus should be evaluated in women at high-risk of preterm birth so as to assess whether accurate visualisation of the gland occurs in the presence of oligohydramnios and whether MRI-derived TV may prove superior in such cases. In addition, exactly how the shape changes in the presence of infection also needs to be evaluated. This is of importance as this subgroup of patients who are at significant risk of fetal infection. Finally, larger studies investigating 2D measurements are representative of true thymus size at different gestational ages should be undertaken.

## Conclusions

Results have demonstrated that thymus volume is a more reproducible measure of thymic size compared to 2D parameters. However, where US is used, the measures that best correlated to thymus volume were 3VV TD, APD and perimeter, making these the more suitable measures in clinical practice where fetal MR scanning may not be feasible due to cost and scanner availability.

## Figures and Tables

**Table 1:** Intraclass Correlation Coefficients for Intra and Inter-reliability for thymic parameters measured on ultrasound scan. 3VV (3 vessel-view), 3VT (3 vessel-trachea view), APD (anterior-posterior diameter), ITD (intrathoracic diameter), TTR (thymic: thoracic ratio), TD (transverse diameter), 3VE (3-vessel-edge). Acceptable ICC values (>0.75 [29]) that are clinically significant (P< = 0.05) are in **bold**.

	Thymus Measurement		Intra-observer reliability (n = 5)			Inter-observer reliability (n = 5)		
			ICC (95% CI)	P value		ICC (95% CI)	P value
	3VV	APD	**0.984** (0.851-0.998)	0.001		**0.913** (0.35-0.99)	0.012	
		ITD	**0.981** (0.860-0.998)	0.001		**0.931** (0.37-0.99)	0.015	
		TTR	**0.903**(0.232-0.990)	0.024		0.771(−0.256-0.968)	0.052	
		TD	**0.954** (0.516-0.995)	0.008		**0.872** (0.700-0.986)	0.035	
		Perimeter	**0.962** (0.648-0.996)	0.002		**0.788** (0.264-0.976)	0.041	
	3VT	APD	**0.894**(0.256-0.989)	0.022		0.731(−0.358-0.782)	0.052	
		ITD	**0.989** (0.917-0.999)	<0.001		0.210(−0.072-0.782)	0.077	
		TTR	**0.996**(0.966-1.000)	<0.001		0.705(0.099-0.963)	0.015	
		TD	**0.852**(−0.200-0.984)	0.049		0.790(−0.590-0.828)	0.375	
		3VE	**0.988** (0.881-0.999)	0.001		**0.901** (−0.191-0.990)	0.032	

**Table 2:** Table showing Pearson Correlation Coefficients (r and r^2^) of 2D ultrasound measurements compared to Thymus Volume (TV). 3VV (3 vessel-view), 3VT (3 vessel-trachea view), APD (anterior-posterior diameter), ITD (intrathoracic diameter), TTR (thymic: thoracic ratio), TD (transverse diameter), 3VE (3-vessel-edge). Clinically significant P values are in **bold**.

Measurement		Thymus Volume (n = 30)		
	*r*	*r* ^2^	P value	
3VV	APD	0.435	0.189	**0.016**
	ITD	0.342	0.117	0.064
	TTR	0.083	0.007	0.664
	TD	0.540	0.292	**0.002**
	Perimeter	0.446	0.199	**0.013**
3VT	APD	0.409	0.167	**0.025**
	ITD	0.325	0.105	0.080
	TTR	0.123	0.015	0.518
	TD	0.544	0.296	**0.002**
	3VE	0.298	0.089	0.110
